# Patient Perspectives on Orthodontic Treatment Challenges, Knowledge, and Attitudes: A Cross-Sectional Survey

**DOI:** 10.7759/cureus.111896

**Published:** 2026-07-01

**Authors:** Sukhpal Kaur, Rashmeet Kaur, Chhaya Mangla, Sameksha Arora, Chitra

**Affiliations:** 1 Orthodontics and Dentofacial Orthopaedics, Desh Bhagat Dental College & Hospital, Mandi Gobindgarh, IND; 2 Periodontology, Desh Bhagat Dental College & Hospital, Mandi Gobindgarh, IND; 3 Periodontology, Maharishi Markandeshwar College of Dental Sciences and Research, Maharishi Markandeshwar Deemed to be University, Ambala, IND; 4 Pediatric and Preventive Dentistry, Santosh Dental College & Hospital, Ghaziabad, IND

**Keywords:** braces, food lodgement, orthodontic treatment, pain, patient attitude, retainer

## Abstract

Background: Orthodontic treatment involves the placement of appliances such as brackets, bands, and wires that are foreign objects within the oral cavity. These appliances, combined with the long treatment duration, can pose various physical and psychological challenges for patients. Understanding these challenges and assessing patient knowledge and attitudes may facilitate targeted patient education and improve treatment compliance.

Methods: A cross-sectional questionnaire-based survey was conducted among 150 patients (aged 15-30 years) undergoing active orthodontic treatment. A structured, pre-validated 20-item questionnaire assessed treatment-related challenges, knowledge, and attitudes toward orthodontic treatment. Data were analyzed using descriptive statistics. Prevalence estimates with 95% confidence intervals (95% CIs) were calculated for all variables.

Results: Food lodgment (54.7%; 95% CI: 46.7%-62.6%) and pain (53.3%; 95% CI: 45.3%-61.3%) were the most frequently reported challenges. Posterior bracket breakage (73.3%) was more frequently reported than anterior bracket breakage (26.7%). The majority of patients demonstrated a positive attitude: 93.3% took extra care in brushing, 86.7% were aware of the need for retainers, and 95.3% would recommend orthodontic treatment to others. Overall treatment satisfaction was high, with 90.6% rating their experience as good or very good.

Conclusion: Food lodgment, pain, and oral ulcers were the most prevalent challenges during orthodontic treatment. Despite these difficulties, patients displayed good knowledge and a positive attitude toward their treatment, highlighting the effectiveness of patient education and counseling.

## Introduction

Malocclusion is among the most common oral health conditions worldwide. It negatively affects dental aesthetics, masticatory function, speech, and psychosocial well-being, often compelling patients to seek orthodontic correction [1,2]. Orthodontic treatment aims to achieve an aesthetically pleasing and functionally stable occlusion by applying controlled forces through various appliances. The appliances used, such as brackets, bands, wires, springs, and elastic chains, are foreign objects placed within the oral cavity for an extended period, which can generate diverse challenges in a patient's daily life [3].

At the outset of treatment, many patients are reluctant, owing to the long treatment duration and the visible presence of orthodontic hardware. Although aesthetic alternatives such as ceramic brackets, lingual systems, and clear aligners have partially addressed this concern, numerous other difficulties persist throughout treatment. Strategies to shorten treatment duration through accelerated tooth movement, including physical, pharmacological, and surgical approaches, have been explored, each carrying its own limitations and indications [4].

Previous studies have identified pain and food lodgment as the most prevalent problems during orthodontic treatment [5]. Pain prevalence has been reported as high as 87%-95% in patients undergoing fixed appliance therapy, particularly in the first 24-48 hours following appliance placement or wire change [6,7]. Beyond pain, fixed orthodontic appliances create plaque-retentive sites that impede oral hygiene, predispose patients to gingival inflammation, and contribute to mucosal irritation, ulceration, and tissue discomfort [8]. Individual responses to these problems vary considerably due to differences in age, pain threshold, appliance type, and psychosocial factors, necessitating systematic investigation to guide clinician counseling.

To date, few studies have comprehensively examined the full spectrum of challenges encountered by orthodontic patients alongside their awareness of and attitude toward treatment [9]. Patient awareness, treatment expectations, and compliance with orthodontic therapy are influenced by sociocultural, educational, and healthcare-system factors, resulting in substantial regional variation in patient experiences and treatment-related behaviors. A recent bibliometric analysis of orthodontic research highlighted marked differences in the geographic distribution of studies investigating patient perceptions, treatment compliance, and oral health awareness, with evidence gaps persisting in several regions despite growing global interest in patient-centered orthodontic care [10]. Understanding both dimensions in a single cohort is important for designing effective pre-treatment education programs. The present study, therefore, aimed to determine the prevalence of treatment-related challenges and to evaluate patient knowledge and attitudes toward orthodontic treatment.

## Materials and methods

A hospital-based cross-sectional survey was conducted among patients receiving fixed orthodontic treatment at the department of orthodontics between April 2025 and April 2026. Assuming a prevalence of 50%, a confidence level of 95%, and a margin of error of 5%, the minimum required sample size was calculated to be 138 participants. Therefore, 150 participants were recruited. Eligible participants were recruited consecutively during routine orthodontic follow-up visits throughout the study period.

Inclusion criteria

The inclusion criteria were patients undergoing orthodontic treatment for the first time, aged 15-30 years, with no history of previous orthodontic treatment, who provided written informed consent, and were physically and mentally healthy.

Exclusion criteria

The exclusion criteria were patients who had previously undergone orthodontic treatment; patients with craniofacial anomalies, syndromes, or congenital dentofacial deformities; patients receiving removable orthodontic appliance therapy only; patients with systemic or psychological conditions that could affect their ability to understand or complete the questionnaire; and questionnaires that were incomplete or inadequately filled.

Questionnaire

A pre-structured, self-administered questionnaire comprising 20 items was used. The first section collected demographic information, including age and gender. The second section assessed treatment-related challenges through 10 items (Table A1), while the third section evaluated patient knowledge, awareness, and attitudes toward orthodontic treatment through 10 items (Table A2). Participants responded based on their own knowledge and treatment experiences. The questionnaire was adapted from previously published studies assessing orthodontic treatment experiences, awareness, and attitudes. Content validity was evaluated independently by three orthodontic specialists, and minor modifications were made based on their recommendations. A pilot test was conducted among 20 orthodontic patients who were not included in the final analysis to assess clarity and comprehensibility.

No additional psychometric validation (e.g., internal consistency testing using Cronbach's alpha or test-retest reliability) was performed after adaptation. Therefore, although the questionnaire was based on previously validated instruments and underwent expert review and pilot testing, the measurement properties of the adapted version were not formally evaluated in the present study. The questionnaire was self-administered in the outpatient clinic and required approximately five to 10 minutes to complete. This estimate was based on the observed completion time during questionnaire administration rather than formal pilot testing.

Statistical analysis

Data were entered into Microsoft Excel (Microsoft Corporation, Redmond, WA) and analyzed by a blinded statistician using IBM SPSS Statistics (version 25.0, IBM Corp., Armonk, NY) and Stata 15 (StataCorp, College Station, TX). Categorical variables were summarized as frequencies and percentages. Prevalence estimates were calculated with corresponding 95% confidence intervals (95% CIs) using the normal approximation method. Missing data were reviewed prior to analysis. Questionnaires with incomplete responses were excluded from the study, and complete-case analysis was performed. Results are presented descriptively.

Ethical approval

This study was approved by the Institutional Ethics Committee of Desh Bhagat Dental College & Hospital, Mandi Gobindgarh, Punjab, India (Reference No. DBU/DBDC/26). The study conforms to the principles outlined in the Declaration of Helsinki. Written informed consent was obtained from all participants prior to their enrolment in the study.

## Results

Participant characteristics

A total of 150 patients undergoing orthodontic treatment participated in the study. The majority were females (n = 110, 73.3%), while males constituted 40 participants (26.7%). Food lodgment was the most frequently reported problem (54.7%; 95% CI: 46.7%-62.6%), followed closely by pain after braces placement (53.3%; 95% CI: 45.3%-61.3%). Oral ulcers were reported by 46.7% (95% CI: 38.7%-54.7%) and difficulty in brushing by 45.3% (95% CI: 37.4%-53.3%) of participants.

**Figure 1:**
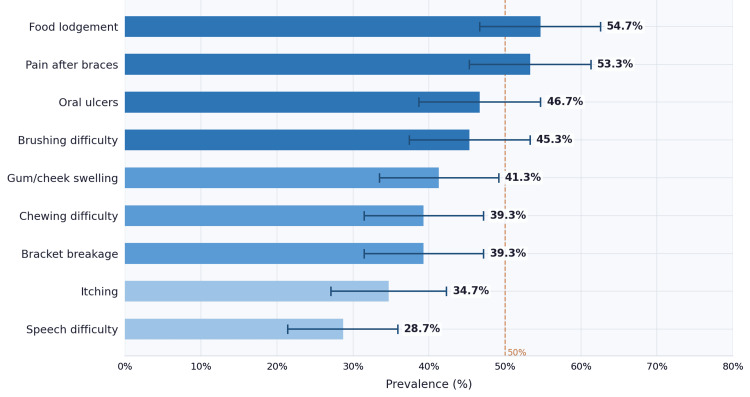
Prevalence of challenges faced during orthodontic treatment (n = 150). Percentages are shown for visual comparison. Detailed prevalence estimates and 95% confidence intervals are reported in the text.

Gingival or cheek swelling was reported by 41.3% (95% CI: 33.5%-49.2%), overall bracket breakage by 39.3% (95% CI: 31.5%-47.2%), and chewing difficulty by 39.3% (95% CI: 31.5%-47.2%). Itching was reported by 34.7% (95% CI: 27.1%-42.3%) and speech difficulty by 28.7% (95% CI: 21.4%-35.9%). Among the 59 patients who reported bracket breakage during treatment, posterior bracket breakage was more common than anterior bracket breakage, accounting for 73.3% (43/59) and 26.7% (16/59) of cases, respectively. The prevalence of treatment-related challenges is illustrated in Figure 1.

Awareness and attitude toward treatment

**Figure 2:**
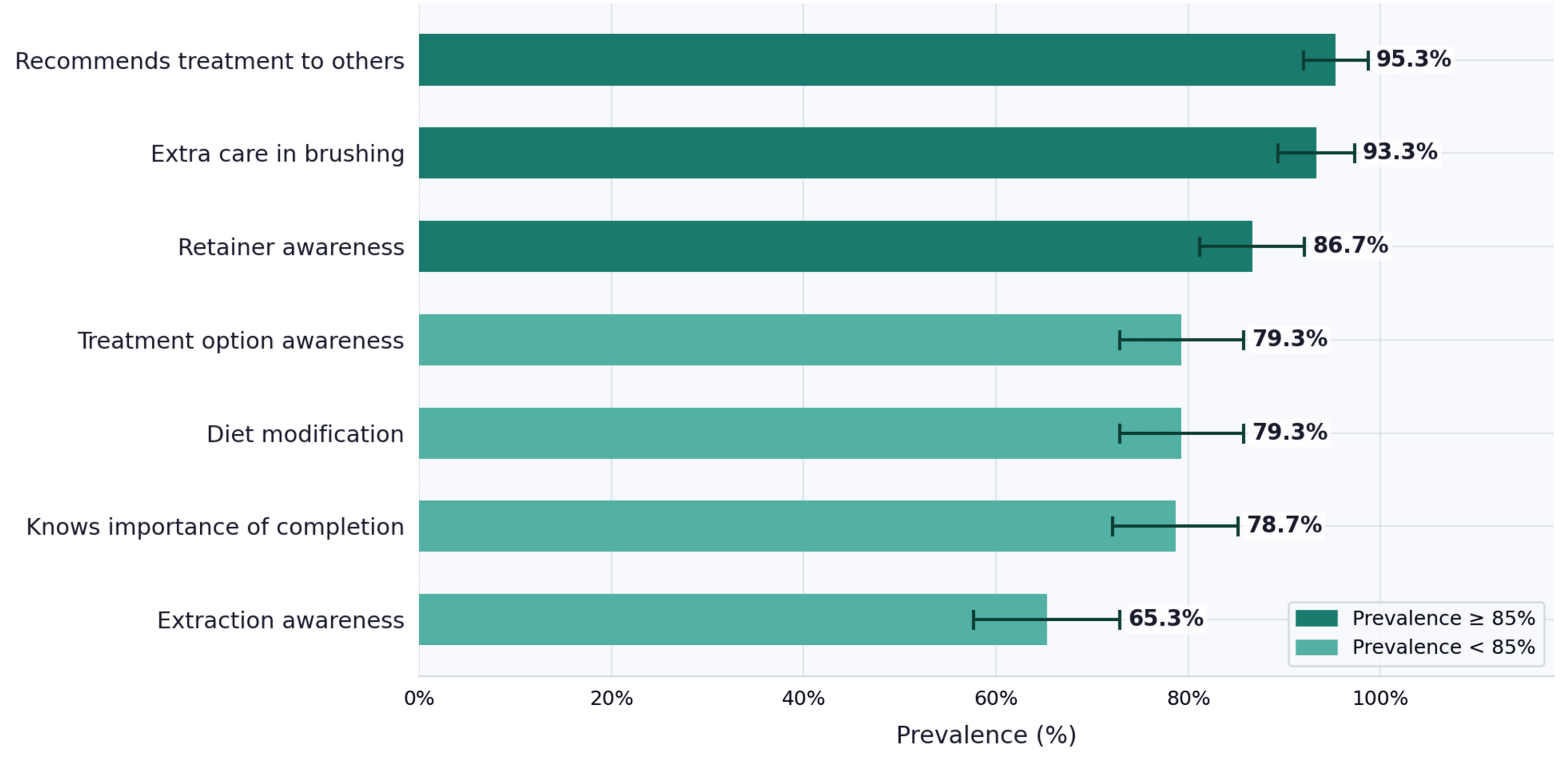
Positive awareness and attitude responses among orthodontic patients (n = 150). Detailed prevalence estimates and 95% confidence intervals are reported in the text.

Patient awareness and attitude were strongly positive across all parameters. The highest prevalence was observed for recommending treatment to others (95.3%; 95% CI: 92.0%-98.7%), extra care in brushing (93.3%; 95% CI: 89.3%-97.3%), and retainer awareness (86.7%; 95% CI: 81.2%-92.1%). Awareness of the importance of treatment completion was reported by 78.7% (95% CI: 72.1%-85.2%), and awareness of different treatment options by 79.3% (95% CI: 72.9%-85.8%). Extraction awareness prior to treatment commencement was present in 65.3% (95% CI: 57.7%-72.9%). Positive awareness and attitude responses are illustrated in Figure 2.

**Figure 3:**
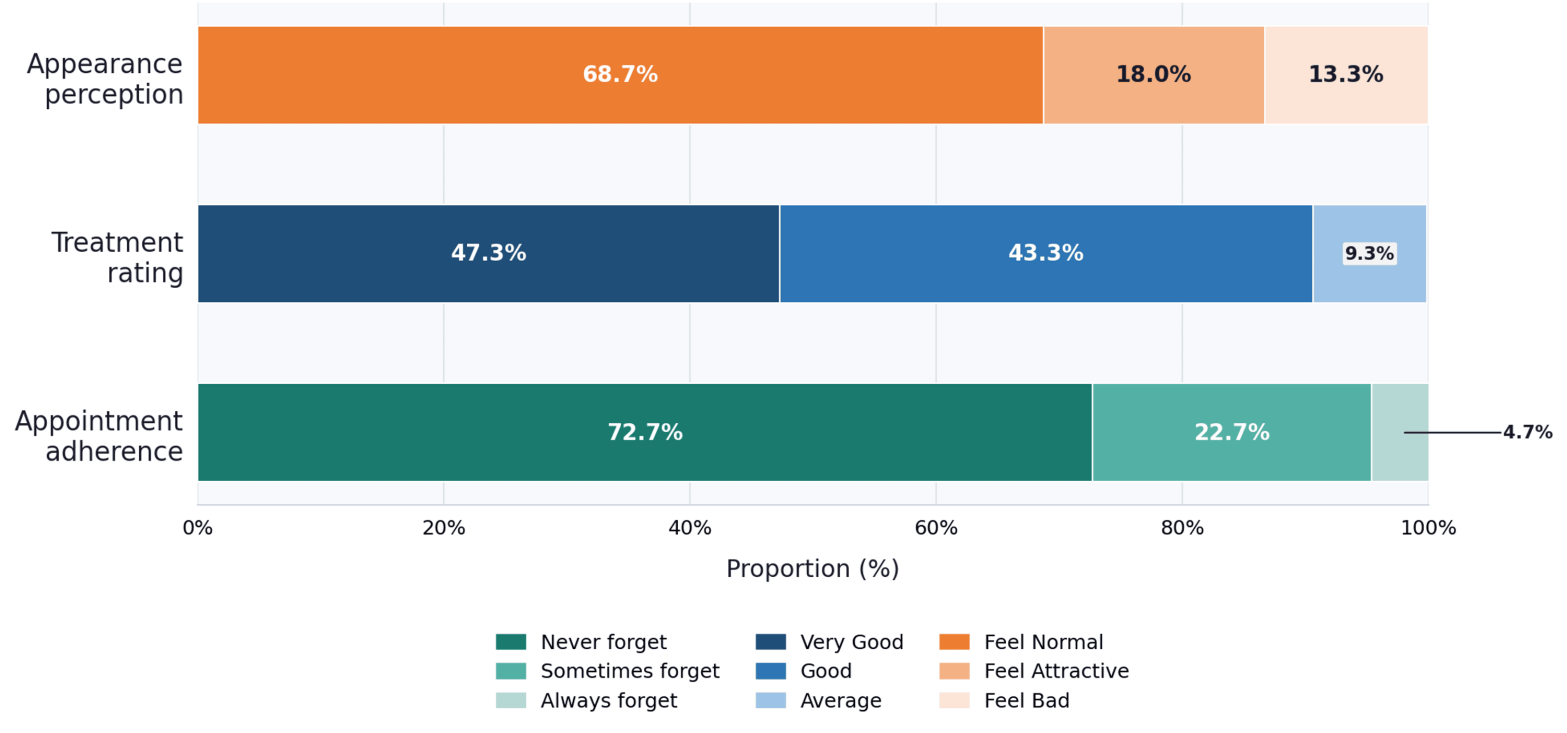
Distribution of treatment satisfaction, appointment adherence, and appearance perception among orthodontic patients (n = 150). Percentages are shown for each response category. Detailed prevalence estimates and 95% confidence intervals are reported in the text.

For multi-category items, 72.7% of patients never forgot appointments (95% CI: 65.5%-79.8%). Treatment satisfaction was high: 47.3% rated their experience as very good (95% CI: 39.3%-55.3%) and 43.3% as good (95% CI: 35.4%-51.3%), with only 9.3% rating it as average. Regarding appearance, 68.7% felt normal (95% CI: 61.2%-76.1%), 18.0% felt attractive, and 13.3% felt bad with their braces. The distribution of treatment satisfaction, appointment adherence, and appearance perception is shown in Figure 3.

## Discussion

This study used a descriptive cross-sectional design to determine the prevalence of challenges experienced by patients undergoing fixed orthodontic treatment and to characterize their knowledge and attitude toward treatment. Reporting prevalence with 95% CIs, rather than applying significance tests within a single group, is the methodologically appropriate approach for this study design and aligns with the purely descriptive objectives.

Food lodgment and pain emerged as the most prevalent challenges, reported by 54.7% and 53.3% of participants, respectively, which is consistent with findings from previous questionnaire-based studies that identified both problems in approximately 50% of orthodontic patients [5]. Pain after appliance placement is a near-universal concern in the orthodontic literature. Studies evaluating pain during the initial phase of fixed orthodontic treatment have reported that severe to moderate pain affects a substantial proportion of patients [7], while prospective investigations comparing conventional, low-friction, and lingual appliance systems have documented pain rates approaching 90% [6]. Ecological momentary assessment studies using smartphone-based monitoring have further demonstrated that pain in adolescent orthodontic patients peaks within the first 24 hours and gradually subsides thereafter [11]. The comparatively lower pain prevalence in the present study (53.3%) likely reflects differences in age group, appliance design, individual pain thresholds, and the timing of questionnaire administration relative to treatment stage. However, the treatment stage was not formally recorded in the present study, and therefore its specific influence on patient-reported outcomes could not be evaluated. Research evaluating pain perception and chewing impairment among adolescents undergoing fixed appliance therapy similarly reported progressive increases in discomfort during treatment [8]. A descriptive cross-sectional study from Nepal reported that 56.6% of orthodontic patients experienced moderate pain, with 88.2% noting painful aching and 83.6% reporting eating difficulty, a pattern broadly consistent with the food lodgment and chewing problems observed in the present cohort [3].

Speech difficulty was reported by only 28.7% of participants in the present study, consistent with previous reports describing a prevalence of approximately 28% [5]. Oral ulcers (46.7%) and chewing difficulty (39.3%), however, were more prevalent in our cohort than previously reported (34% and 29%, respectively), possibly reflecting differences in appliance type, bracket design, and dietary habits. Comparative studies evaluating different orthodontic appliance systems have demonstrated that appliance type is a key determinant of pain and discomfort experience, with lingual appliance patients reporting lower pain levels than those treated with conventional brackets or clear aligners [6]. A recent pilot study evaluating pain perception, knowledge, attitude, and dietary diversity among orthodontic patients found that approximately 90% perceived low pain levels and that good knowledge scores were positively correlated with a favorable attitude toward treatment, a finding that aligns with the high awareness and satisfaction levels observed in the present study [9].

Among the 59 patients who reported bracket breakage, posterior breakage was considerably more prevalent than anterior breakage (73.3% vs. 26.7%). This finding is clinically plausible, as molar and premolar brackets are subjected to greater masticatory forces, moisture contamination during bonding is more likely in the posterior segments, and access for adequate light-curing is more restricted. This pattern has practical implications for chairside technique, particularly in ensuring optimal enamel preparation and adhesive application in the posterior dentition.

Retainer awareness in the present study (86.7%; 95% CI: 81.2%-92.1%) was notably high compared with ranges of 45.7%-79.9% reported in prior surveys [12,13]. Previous questionnaire-based studies documented retainer awareness of approximately 79.9% and dietary modification in 59.6% of patients, both lower than our findings of 86.7% and 79.3%, respectively [12]. The higher rates observed in the present study may reflect differences in patient characteristics, clinical practices, or other unmeasured factors. However, these variables were not specifically evaluated, and therefore no causal inferences can be drawn. Oral hygiene vigilance was also high at 93.3%, exceeding previously reported rates of approximately 82% [12]. Studies assessing periodontal health awareness among orthodontic patients have demonstrated that awareness is significantly influenced by age, attitude, and treatment duration, highlighting that knowledge develops throughout treatment [14]. Other questionnaire-based investigations have similarly emphasized that proactive, structured oral hygiene counseling is essential for improving patient compliance with periodontal health maintenance during fixed orthodontic treatment [13,15]. Surveys conducted among North Indian orthodontic populations have also reported good patient knowledge regarding retainer wear and treatment completion, suggesting that regional patient populations may share baseline literacy regarding orthodontic requirements [16].

Overall treatment satisfaction was high, with 90.6% of patients rating their experience as good or very good. This is comparable to findings from studies evaluating treatment satisfaction among adult orthodontic patients, in which the majority reported being satisfied or greatly satisfied with treatment outcomes [17]. Awareness of the possibility of extraction as part of treatment was present in 65.3% of participants prior to commencing treatment, consistent with findings from similar questionnaire-based surveys reporting rates of approximately 61% [18]. This figure was considerably higher than that reported in other cross-sectional surveys (33.5%), a discrepancy likely attributable to variation in pre-treatment counseling protocols and the educational background of patient populations across different centers [19]. The very high recommendation rate (95.3%) and the low proportion of patients who felt bad about their appearance with braces (13.3%) suggest an overall positive perception of treatment among participants. However, the factors contributing to these perceptions were not specifically investigated and warrant further study.

Limitations

This study has several limitations that should be considered when interpreting the findings. First, its cross-sectional design precludes assessment of temporal changes in patient experiences, knowledge, and attitudes throughout the course of orthodontic treatment. Second, the data were obtained through self-reported questionnaires and are therefore subject to recall bias, response bias, and social desirability bias. Third, patients were not stratified according to the stage of orthodontic treatment, which may have influenced the prevalence and severity of treatment-related challenges, particularly pain and discomfort. Fourth, educational background and health literacy were not assessed, limiting the evaluation of their potential influence on patient knowledge and attitudes toward orthodontic treatment. The study was also conducted at a single center with a moderate sample size, which may limit the generalizability of the findings to other populations and healthcare settings. In addition, although the questionnaire was adapted from previously validated instruments and underwent expert review and pilot testing, no formal psychometric evaluation (e.g., internal consistency or reliability testing) of the adapted version was performed, which may have affected its measurement properties in the present study. Finally, subgroup analyses based on age, sex, treatment duration, or socioeconomic status were not performed. Future multi-center longitudinal studies incorporating treatment-stage stratification and educational variables would provide a more comprehensive understanding of factors influencing patient experiences and treatment awareness.

Clinical implications

Identifying and addressing challenges during orthodontic treatment enables clinicians to counsel patients proactively. Specific guidance on brushing techniques, use of interdental aids, and dietary modification can minimize food lodgment and bracket breakage. Targeted pre-treatment education regarding retainer compliance, treatment completion, and oral hygiene management can enhance patient compliance and satisfaction, ultimately improving treatment outcomes.

## Conclusions

Food lodgment, pain, and oral ulcers were the most prevalent challenges reported by orthodontic patients in this study. Despite these difficulties, the cohort demonstrated good knowledge and a positive attitude toward treatment, including high awareness of retainers, treatment completion, and oral hygiene requirements. These findings highlight the generally positive levels of awareness and attitudes observed among participants and support the potential value of effective patient education and counseling during orthodontic treatment. Further studies are needed to evaluate the specific impact of these interventions on patient experiences and treatment outcomes.

## Appendices

Table A1

**Table 1:** Questionnaire items used to assess challenges experienced during orthodontic treatment.

S. No.	Question	Response options
1	Do you experience difficulty in brushing your teeth with braces?	Yes / No
2	Do you experience pain after placement of braces and at every visit when the wire is changed?	Yes / No
3	Do you experience frequent ulcers in the mouth after braces?	Yes / No
4	Do you experience any problems in speaking with braces?	Yes / No
5	Do you experience any problems in chewing with braces?	Yes / No
6	Do you experience frequent food lodgment in your teeth with braces?	Yes / No
7	Do you experience any swelling in your gums or cheeks after braces?	Yes / No
8	Do you feel itching after placement of braces in your mouth, gums, or cheeks?	Yes / No
9	Do you experience frequent bracket breakage during treatment?	Yes / No
10	In which area do you commonly experience bracket breakage?	(a) Forward (b) Backward

Table A2

**Table 2:** Questionnaire items used to assess patient awareness and attitudes toward orthodontic treatment.

S. No.	Question	Response options
1	Do you take extra care in brushing to clean your teeth after wearing braces?	Yes / No
2	Did you modify your dietary habits after the placement of braces?	Yes / No
3	Do you know that extractions (removal of teeth) might be needed during orthodontic treatment?	Yes / No
4	How do you feel about your appearance with braces?	(a) Feel bad (b) Feel attractive (c) Feel normal
5	Will you recommend orthodontic treatment to others?	Yes / No
6	Do you know about the different types of treatment options in orthodontics?	Yes / No
7	Do you know the importance of wearing retainers after treatment?	Yes / No
8	Do you know the importance of completing treatment? Otherwise, the results will not be good.	Yes / No
9	How would you rate your orthodontic treatment?	(a) Average (b) Good (c) Very good
10	Do you forget your appointments with your orthodontist?	(a) Yes (b) Sometimes (c) No
